# Sustainability of a rehabilitation self‐management program (‘My Therapy’) 6 months post implementation

**DOI:** 10.1111/1440-1630.70030

**Published:** 2025-06-09

**Authors:** Sara L. Whittaker, Keith D. Hill, Nicholas F. Taylor, Christina L. Ekegren, Natasha K. Brusco

**Affiliations:** ^1^ Rehabilitation, Ageing and Independent Living (RAIL) Research Centre, School of Primary and Allied Health Care Monash University Melbourne Victoria Australia; ^2^ School of Allied Health, Human Services and Sport La Trobe University Melbourne Victoria Australia; ^3^ Eastern Health, Allied Health Clinical Research Office Melbourne Victoria Australia; ^4^ Alfred Health Melbourne Victoria Australia; ^5^ Cabrini Health Melbourne Victoria Australia; ^6^ School of Public Health and Preventive Medicine Melbourne Victoria Australia

**Keywords:** occupational therapy, physiotherapy, rehabilitation, self‐management

## Abstract

**Introduction:**

The aim of this study was to explore sustainability of a self‐management program in inpatient rehabilitation (‘My Therapy’) 6 months following a randomised controlled trial.

**Methods:**

A patient audit of rehabilitation hospital medical records was completed to determine program reach and an electronic survey of occupational therapists/physiotherapists to understand perceptions of program sustainability.

**Consumer and community involvement:**

This study included a lived experience consumer as part of the trial steering committee.

**Results:**

Of 185 patients audited, the program reach was 41%. Of 13 therapists surveyed, most reported they knew how to deliver self‐management programs (93%), that they provided My Therapy as part of usual care ‘some of the time’ (77%), and that appointing a My Therapy clinical champion on the ward supported sustained implementation (77%). They also reported reduced confidence delivering ‘My Therapy’ with patients who were not motivated or when time was limited.

**Conclusion:**

Six months post‐trial, a self‐management program was still being delivered by therapists in rehabilitation. Ongoing support strategies are required to sustain self‐management programs.

**PLAIN LANGUAGE SUMMARY:**

Doing exercises and practising tasks is an important part of rehabilitation. A self‐management program called ‘My Therapy’ enables patients to do exercises or tasks on their own. Patients doing My Therapy completed an extra 26 minutes of exercises or tasks each day. This study in Melbourne, Australia, found out if the program was still being used 6 months later. We also found out what therapists thought about the program. Less than half of patients were doing My Therapy at 6 months. Therapists reported they liked their patients doing the program. However, they found it harder to deliver to patients who lacked motivation. It was also hard to deliver when the therapist felt they had little time.

**Key Points for Occupational Therapy**

- Occupational therapists and physiotherapists can increase therapy participation through facilitation and support of patient self‐management.
- Six months after trial completion, ‘My Therapy’ was delivered to fewer than half of patients.
- For lower‐motivated patients, support strategies/resources for therapists could be required to improve delivery of ‘My Therapy’.

## 1 INTRODUCTION

Self‐management programs can be delivered in rehabilitation settings at minimal cost and may improve patients' health‐related quality of life (Whittaker, Brusco, Hill, & Taylor, 2024). Self‐management programs require the active participation of a person in rehabilitation and could involve them practising activities and exercises to improve skills and functional performance on their own (Fryer et al., 2016). Self‐management programs have been shown to be effective in improving clinical outcomes for many clinical populations, such as stroke and osteoarthritis (Devos‐Comby et al., 2006; Fryer et al., 2016). One example of a self‐management program suitable for rehabilitation settings is the My Therapy program, which was recently evaluated for its effectiveness and cost‐effectiveness in a stepped wedge cluster randomised controlled trial (Brusco et al., 2021; Brusco et al., 2024). Within the trial, My Therapy programs included a sub‐set of activities prescribed by an occupational therapist and/or physiotherapist that the patient was able to complete safely and independently without health professional supervision (for further details, see Data S1). My Therapy was implemented alongside usual care on participating rehabilitation wards with occupational therapists and physiotherapists requiring a change of clinical practice for therapists. To be considered self‐management, the My Therapy program met the following criteria: (i) written; (ii) recorded in the participant's medical record; (iii) monitored and progressed each week or as clinically required; (iv) feedback between participant and clinician (e.g., via an activity recording template) (Brusco et al., 2024; Whittaker, Brusco, Hill, Ekegren, & Taylor, 2024). The intention of the My Therapy program was that occupational therapists and physiotherapists supported the development and review of the My Therapy program, with the patient being able to choose when and how much of their My Therapy program they did outside of supervised therapy (Brusco et al., 2024).

Implementing My Therapy programs required behaviour change from the therapists on participating wards, which was supported through staff training and mentorship from an on‐site study coordinator. Although immediate behaviour change was apparent during the trial, with My Therapy reaching 68% of patients on the ward (Whittaker, Brusco, Hill, Ekegren, & Taylor, 2024), it is unknown whether these changes were sustained following the trial. This information is important for understanding the factors associated with long‐term sustainability of self‐management programs within rehabilitation settings. The aim of this study was to explore sustainability and therapists' experiences of implementing a self‐management program (My Therapy) 6 months following the conclusion of the formal program evaluated in a clinical trial.

## 2 METHODS

### 2.1 Study design and setting

This study, completed 6 months after the conclusion of the stepped wedge randomised control trial of My Therapy, was conducted across eight rehabilitation wards at two public and two private hospitals in Victoria, Australia (Brusco et al., 2021). Alfred Hospital Human Research Ethics Committee (HREC) (ID: 69610) provided multi‐site ethics approval, followed by site specific approvals of each of the participating health services (Alfred Hospital, ID 758/20; Eastern Health, ID S21‐004‐69610; Cabrini Health, ID 11‐04‐03‐21; Healthscope via La Trobe HREC, ID 758/20).

### 2.2 Implementation of ‘My Therapy’

During the main trial, planning for My Therapy implementation occurred 6 weeks prior to wards crossing over to intervention conditions, when self‐management programs were then implemented as part of usual care. During the 6‐week pre‐implementation period, clinical staff (occupational therapists and physiotherapists) received education and written resources supporting the implementation of the intervention and participated in group discussions to co‐design local implementation strategies (Whittaker, Brusco, Hill, Ekegren, & Taylor, 2024). Following the trial, participating wards intended to continue delivering My Therapy self‐management as part of usual care, within their usual clinical workload. Participating wards indicated their ongoing implementation of My Therapy through the completion of an audit post‐trial conclusion. To support ongoing implementation, My Therapy training using existing resources for new staff was embedded into local practices.

### 2.3 Participants

There were two groups of participants 6 months after completion of the trial: (i) all patients on the eight participating wards on the day of audit; and (ii) occupational therapists and physiotherapists therapists delivering clinical care on wards who participated in the clinical trial (October 2022). Consent for patient participants was waived by the ethics committee, as only de‐identified routine data were collected. The only eligibility criterion for My Therapy was that the patient had been admitted to a participating ward; reasons for non‐participation were documented. Therapist consent was implied via survey completion.

### 2.4 Data collection tools

For patients, an audit (Data S2) was completed on a single day 6 months following trial conclusion. The trial concluded in April 2022, with the audit completed on a day nominated by the participating ward during the month of October, 2022 (one health service due to staffing constraints completed on a day at the end of September 2022). The audit captured the prescription (or not) of a My Therapy program, and the model of care under which the patient was admitted (specifically ‘rehabilitation’ or ‘geriatric evaluation and management’ [GEM, noting GEM is a specialised sub‐acute service associated with medical conditions related to ageing [Victorian Department of Health, 2022]). To be included in the audit, the My Therapy program needed to meet criteria detailed earlier (Whittaker et al., 2021). Data were collected at each participating site on a REDCap form (Research Electronic Data Capture) hosted by Monash University and managed by Helix (Harris et al., 2009; Harris et al., 2019). For therapists, a customised REDCap survey (Data S3) presented in a mix of open format and Likert‐scale questions was emailed to therapists working on participating wards. The survey was designed to explore the barriers and enablers of continued implementation of My Therapy.

### 2.5 Data analysis

Implementation reach of My Therapy was reported as the percentage of patients who were prescribed with a My Therapy program (i.e., the number of patients with a My Therapy program divided by the number of patients admitted to the ward). For categorical survey data, responses were recorded as the percentage of therapists who agreed with each statement. For survey responses based on Likert scales (0 to 100), the median, inter‐quartile range (IQR), and range were reported. The Likert scale anchors varied depending on the question type, for example, for some responses a score of 0 meant being ‘Not at all confident’ and 100 being ‘Very confident’.

### 2.6 Author positionality statements

Dr Sara Whittaker is an occupational therapist and researcher with clinical experience in rehabilitation. She led this research study, including methodological design, data collection, data analysis, and reporting of the results. Professor Keith Hill is a physiotherapist, academic, and researcher specialising in fall prevention, exercise for older people, ageing well, and rehabilitation. Professor Nicholas Taylor is a physiotherapist, academic, and researcher specialising in research focused on the role of exercise and physical activity for people with disability or injuries and how to provide health services for rehabilitation. Associate Professor Christina Ekegren is a physiotherapist, academic, and researcher specialising in physical activity in clinical populations, specifically hospitalised patients, older adults, and people recovering from traumatic injury. Associate Professor Natasha Brusco is a physiotherapist, academic, and researcher pioneering new ways to evaluate cost effective models of care, which included My Therapy. Throughout the study, including the analysis, authors reflected on their positionality, which included having interdisciplinary professional perspectives (occupational therapy and physiotherapy).

## 3 RESULTS

### 3.1 Participants

A total of 185 patients were included in the ward audit, with 106 admitted for rehabilitation and 79 admitted for GEM. The number of admitted patients on the eight participating wards ranged from 12 to 30 (median 26). Thirteen therapists (of the potential 40) completed surveys, including five occupational therapists (Table 1). Twelve of these therapists were employed by the health service during the preceding clinical trial.

**TABLE 1:** Therapist participant demographics (*n* = 13).

	Therapists *n* (%)
Profession	
Occupational therapist	5 (39%)
Physiotherapist	8 (61%)
Gender	
Female	11 (85%)
Male	2 (15%)
Years of clinical experience	
<1 year	1 (8%)
1 to 4 years	3 (23%)
5 to 14 years	5 (38%)
≥15 years	4 (31%)
Health service	
Health service 1	1 (8%)
Health service 2	5 (38%)
Health service 3	6 (46%)
Health service 4	1 (8%)

### 3.2 Implementation reach of My Therapy at 6 months

Forty‐one percent of patients (*n* = 75/182, *n* = 3 missing data) had a My Therapy program prescribed on the day of audit. Of the 92% of patients (*n* = 168) who received occupational therapy, 14% (*n* = 24/168) were prescribed a My Therapy program by the occupational therapist. Of the 97% of patients (*n* = 176) who received physiotherapy input, 35% (*n* = 62/176) were prescribed a My Therapy program by the physiotherapist. Fourteen patients who were a new admission to the ward or too early in their admission to have received a My Therapy program were included in the analysis.

### 3.3 Therapists' experience of My Therapy at 6 months

**FIGURE 1:**
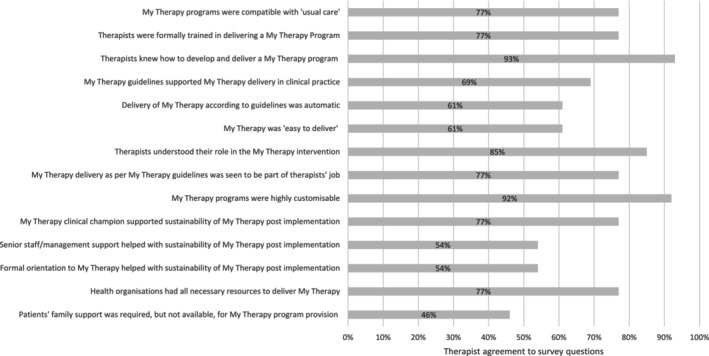
Percentage of therapists agreeing to each statement (*n* = 13).

At 6 months, 77% of therapists reported they provided patients with My Therapy programs as part of usual care ‘some of the time’ (*n* = 10/13), one therapist reported they did this ‘all of the time’, one therapist reported they did it ‘rarely’, and one therapist reported they did it ‘none of the time’. Most therapists agreed that My Therapy was compatible with ‘usual care’, and that they had received formal training to deliver My Therapy (Figure 1). Most therapists agreed that they knew how to develop and deliver a self‐management program, with the My Therapy guidelines guiding program development in clinical practice. Despite this, fewer therapists agreed that delivering My Therapy was something they did automatically, and most agreed it was easy to deliver. Most therapists agreed that My Therapy programs were highly customisable and they had the required resources within their organisation. Less than half the therapists agreed that patient's family support was required but not available. Therapists agreed that a number of strategies helped sustain My Therapy on the ward, including the role of a My Therapy clinical champion on the ward and senior staff/management support to provide self‐management programs and formal orientation to My Therapy.

Table 2 provides the responses (0–100) for a number of survey statements. There was a high agreement that delivering My Therapy was worthwhile. High confidence levels were reported for delivering My Therapy and continuing to do this even when other professionals were not. Therapists reported lower levels of confidence delivering My Therapy with patients who were not motivated or when there was little time. There was a high level of agreement that My Therapy programs helped patients to be more physically active but only moderate agreement for whether patients were motivated. Therapists placed a lower level of importance on My Therapy programs, compared with supervised programs.

**TABLE 2:** Therapist responses to survey questions, on a 0–100 scale (*n* = 13).

Survey question	Median (IQR)	Range
Level of confidence (not at all confident to very confident)		
Do you feel confident that you have the skills to deliver My Therapy following the guidelines?	80.0 (74.5–90.5)	55.0–95.0
Do you feel confident that you can deliver My Therapy following the guidelines even when other professionals with whom you work with do not do this?	79.0 (64.0–89.5)	50.0–99.0
Do you feel confident that you can deliver My Therapy following the guidelines even when there is little time?	55.0 (44.0–71.0)	24.0–83.0
Do you feel confident that you can deliver My Therapy following the guidelines even when patients are not motivated?	50.0 (38.0–56.5)	0.0–86.0
Level of worth (not at all worthwhile to very worthwhile)		
Please respond to this statement: For me, delivery My Therapy following the guidelines is:	74.0 (50.0–86.0)	35.0–89.0
Level of agreement (strongly disagree to strongly agree)		
To what extent do you agree with the following statement: My Therapy programs help patients to be more physically active.	75.0 (58.5–86.0)	50.0–94.0
To what extent do you agree with the following statement: Patients who receive a My Therapy program are motivated.	50.0 (50.0–60.0)	41.0–74.0
Level of importance (much less important to much more important)		
Compared to supervised therapy sessions, how important are My Therapy programs?	40.0 (33.0–50.0)	27.0–62.0

Via free‐text responses, therapists reported a number of barriers to the sustainability of implementing self‐management programs following trial conclusion. These included changeover of staff requiring new orientation to My Therapy; difficulties navigating the chosen online exercise platform to generate programs; complexity and time taken to develop programs addressing activity and participation limitations (such as dressing tasks), compared with body and structure limitations (such as strengthening exercises); clinical time availability; and patient motivation to participate in My Therapy. Three therapists had concerns regarding patient safety, particularly for patients with cognitive impairment or clinical precautions.

## 4 DISCUSSION

At 6 months post‐trial completion, about 4 in 10 patients received a My Therapy program, consistent with most therapists reporting that My Therapy was implemented some of the time. During the clinical, trial My Therapy was delivered to 68% of patients on participating wards, increasing therapy dosage by 26 minutes per day (Whittaker, Brusco, Hill, Ekegren, & Taylor, 2024). The results of the current study suggest that additional support strategies should be considered to improve sustained implementation of self‐management programs to rehabilitation patients by occupational therapists and physiotherapist with the aim to increase therapy dosage.

Support strategies to sustain implementation of self‐management programs may include conducting regular audits of program prescription for monitoring and feedback to therapists. In addition, targeted strategies could be used to help reach patients with cognitive impairments or safety concerns. For these patients, memory support strategies in the form of visual and auditory prompts and external supports (such as visitors, i.e., family and friends) could be helpful. This study suggests that therapists could benefit from education regarding strategies that might help to motivate patients to participate in self‐management activities. For patient's receiving My Therapy, it was critical for therapists to understand the driving and limiting factors for uptake of My Therapy participation, and for My Therapy, it was reported that many of the driving forces were linked to pre‐existing personal traits, values, and goals (Dorward et al., 2024). More broadly, strategies to improve patient's motivation to complete self‐management could include setting relevant and personalised goals, providing education and information about the program and understanding the patient's beliefs or cultures (Maclean et al., 2002). Interestingly, therapists rated My Therapy as almost equal importance to supervised therapy and therefore increasing patient participation would be of value. It is recognised that the time spent to develop and provide a My Therapy program is completed within existing staffing resources. Providing My Therapy may take extra time for the more complex patients meaning that within limited resources, therapists may be prioritising My Therapy provision with the more straightforward patients. The provision of a My Therapy program is likely to require therapist behaviour change and re‐prioritisation of clinical demands that will need to be supported by key stakeholders within the organisation.

Although all patients admitted on the day of the audit were included, which is a strength of this study, a key limitation of this study is that it only about 30% of the therapists working on participating wards completed the surveys, which could reflect responder bias. However, there was representation across all four participating health services potentially increasing the generalisability of study findings. The lack of formal pilot testing of the survey is a limitation. We adopted a pragmatic approach by piloting with members of the steering committee prior to the start of the trial.

## AUTHOR CONTRIBUTIONS

Author SW took the lead in writing the manuscript. All authors provided critical feedback and helped shaped the research, analysis and manuscript.

## CONFLICT OF INTEREST STATEMENT

The authors declare that they have no competing interests.

## Supporting information


**Data S1:** Tidier Checklist of My Therapy intervention.
Data S2: Patient Audit.
Data S3: Staff Survey

## Data Availability

The data that support the findings of this study are available from the corresponding author upon reasonable request.

## References

[aot70030-bib-0001] Brusco, N. K. , Ekegren, C. L. , Morris, M. E. , Hill, K. D. , Lee, A. L. , Somerville, L. , Lannin, N. A. , Abdelmotaleb, R. , Callaway, L. , Whittaker, S. L. , & Taylor, N. F. (2024). Outcomes of the My Therapy self‐management program in people admitted for rehabilitation: A stepped wedge cluster randomized clinical trial. Annals of Physical and Rehabilitation Medicine, 67(8), 101867. 39173328 10.1016/j.rehab.2024.101867

[aot70030-bib-0002] Brusco, N. K. , Ekegren, C. L. , Taylor, N. F. , Hill, K. D. , Lee, A. L. , Somerville, L. , Lannin, N. A. , Wade, D. , Abdelmotaleb, R. , Callaway, L. , Whittaker, S. L. , & Morris, M. E. (2021). Self‐managed occupational therapy and physiotherapy for adults receiving inpatient rehabilitation (‘My Therapy’): Protocol for a stepped‐wedge cluster randomised trial. BMC Health Services Research, 21(1), 811. 10.1186/s12913-021-06462-9 34384427 PMC8361638

[aot70030-bib-0003] Devos‐Comby, L. , Cronan, T. , & Roesch, S. C. (2006). Do exercise and self‐management interventions benefit patients with osteoarthritis of the knee? A metaanalytic review. The Journal of Rheumatology, 33(4), 744–756. 16583478

[aot70030-bib-0004] Dorward, E. , Devlin, A. , Brusco, N. K. , Dulfer, F. , Whittaker, S. L. , Reeder, S. , & Ekegren, C. L. (2024). Patients' perceptions of participating in self‐directed activities outside supervised occupational and physiotherapy within inpatient and home‐based rehabilitation settings: A qualitative study. Disability and Rehabilitation, 47(3), 592–600. 10.1080/09638288.2024.2341872 38625404

[aot70030-bib-0005] Fryer, C. E. , Luker, J. A. , McDonnell, M. N. , & Hillier, S. L. (2016). Self management programmes for quality of life in people with stroke. Cochrane Database of Systematic Reviews, 2016(8), CD010442. 10.1002/14651858.CD010442.pub2 27545611 PMC6450423

[aot70030-bib-0006] Harris, P. A. , Taylor, R. , Minor, B. L. , Elliott, V. , Fernandez, M. , O'Neal, L. , McLeod, L. , Delacqua, G. , Delacqua, F. , Kirby, J. , Duda, S. N. , & REDCap Consortium . (2019). The REDCap consortium: Building an international community of software platform partners. Journal of Biomedical Informatics, 95, 103208. 10.1016/j.jbi.2019.103208 31078660 PMC7254481

[aot70030-bib-0007] Harris, P. A. , Taylor, R. , Thielke, R. , Payne, J. , Gonzalez, N. , & Conde, J. G. (2009). Research electronic data capture (REDCap)—A metadata‐driven methodology and workflow process for providing translational research informatics support. Journal of Biomedical Informatics, 42(2), 377–381. 10.1016/j.jbi.2008.08.010 18929686 PMC2700030

[aot70030-bib-0008] Maclean, N. , Pound, P. , Wolfe, C. , & Rudd, A. (2002). The concept of patient motivation. Stroke, 33(2), 444–448. 10.1161/hs0202.102367 11823650

[aot70030-bib-0009] Victorian Department of Health . (2022). Geriatric evaluation and management. https://www.health.vic.gov.au/patient-care/geriatric-evaluation-and-management

[aot70030-bib-0010] Whittaker, S. L. , Brusco, N. K. , Hill, K. D. , Ekegren, C. L. , & Taylor, N. F. (2024). A self‐management program increases the dosage of inpatient rehabilitation by 26 minutes per day: A process evaluation. Disability and Rehabilitation, 47(2), 425–434. 10.1080/09638288.2024.2339533 38627962

[aot70030-bib-0011] Whittaker, S. L. , Brusco, N. K. , Hill, K. D. , & Taylor, N. F. (2024). Self‐management programs within rehabilitation yield better health outcomes at a small increased cost: A systematic review and meta‐analysis. Archives of Physical Medicine and Rehabilitation, 105(10), 1946–1960. 38729404 10.1016/j.apmr.2024.05.007

[aot70030-bib-0012] Whittaker, S. L. , Taylor, N. F. , Hill, K. D. , Ekegren, C. L. , & Brusco, N. K. (2021). Self‐managed occupational therapy and physiotherapy for adults receiving inpatient rehabilitation (‘My Therapy’): Protocol for a mixed‐methods process evaluation. BMC Health Services Research, 21(1), 810. 10.1186/s12913-021-06463-8 34384420 PMC8361854

