# Supplementary material for: Sustainability of a rehabilitation self‐management program (‘My Therapy’) 6 months post implementation
Source: Aust Occup Ther J. 2025 Jun 9;72(3):e70030. doi: 10.1111/1440-1630.70030 (PMC12149698; doi:10.1111/1440-1630.70030)
Supplement: Supplementary file 1 — Data S1: Tidier Checklist of My Therapy intervention. Data S2: Patient Audit. Data S3: Staff Survey [file AOT-72-0-s001.pdf]

Supplementary Materials

Table of Contents

Supplementary file 1: Tidier Checklist of My Therapy intervention 2

Supplementary file 2: Patient Audit 4

Supplementary file 3: Staff Survey 8

Supplementary file 1: Tidier Checklist of My Therapy intervention

Description of control and intervention conditions, in accordance with the template for intervention description and replication (TIDieR)

	Control	Intervention
Brief name	Usual care rehabilitation	My Therapy in addition to usual care rehabilitation
Why	To provide a comparison with My Therapy	A higher dose of inpatient rehabilitation may result in better participant and health service outcomes
What materials	Site-dependent, in accordance with usual care rehabilitation. May include exercise equipment and equipment to facilitate activities of daily living.	Participants: Paper-based My Therapy program with exercise and activity recording template in multiple languages; exercise equipment as required, such as light hand weights.<br>Staff: Database of occupational therapy and physiotherapy activities and exercises, e.g. PTX online program, or other online programs or existing paper-based collections available within the hospital, if preferred.
What procedures	Occupational therapists and physiotherapists complete an assessment, set goals and develop a rehabilitation program.	Where deemed safe and appropriate, a sub-set of the supervised activities and exercises from participants' rehabilitation program provided to participants to be practiced independently, outside of supervised sessions. My Therapy programs should: i) be in written format; ii) be recorded in the participant's medical record; iii) be actively monitored and progressed, as clinically required; and iv) include a feedback mechanism between participant and clinician (e.g. via the My Therapy exercise and activity recording template).
Who provides	Activities and exercises completed by participants under the supervision of registered occupational therapists and physiotherapists employed by the hospital.	Programs developed by occupational therapists and physiotherapists and completed by participants independently without supervision or assistance. Occupational therapists and physiotherapists encouraged to collaborate to provide a combined program to participants.

How provided	Provided in-person, within scheduled sessions.	Program adherence reinforced with verbal reminders from the multi-disciplinary clinical team, including allied health assistants, the use of blue folders which contained participants' programs in paper format and were strategically placed in visible and convenient locations in their rooms, the provision of My Therapy brochures for participants and their next of kin, and the placement of posters around the ward.
Where (setting)	Often in gyms or therapy rooms within rehabilitation hospitals, or in the home.	Participants' rooms on the ward, the hallway, or other designated independent practice areas within rehabilitation hospitals, or in the home.
When/how much (dose)	Most commonly, daily occupational therapy and physiotherapy sessions, one hour in duration, from Monday to Friday. Additional daily sessions, group sessions and weekend sessions site-dependent, based on staff ratios and funding	Dose prescribed by treating occupational therapist and physiotherapist according to participant goals.
Tailoring	Activities and exercises tailored for each participant according to their rehabilitation goals and progressed throughout the rehabilitation admission.	Activities and exercises tailored for each participant according to their rehabilitation goals and progressed throughout the rehabilitation admission.
Fidelity checking measures	Adherence and fidelity assessed within a comprehensive process evaluation.	As per usual care + completion of a My Therapy exercise and activity recording template by the participant.

Sourced from:

Brusco, N. K., Ekegren, C. L., Morris, M. E., Hill, K. D., Lee, A. L., Somerville, L., Lannin, N. A., Abdelmotaleb, R., Callaway, L., Whittaker, S. L., & Taylor, N. F. (2024). Outcomes of the My Therapy self-management program in people admitted for rehabilitation: A stepped wedge cluster randomized clinical trial. *Annals of Physical and Rehabilitation Medicine*, *67*(8), 101867. https://doi.org/https://doi.org/10.1016/j.rehab.2024.101867

Supplementary file 2: Patient Audit

Process Evaluation Ward Audit

Record ID ______________________

Ward Audit

Instructions:
The ward audit is completed each block of My Therapy during Week 3.

It is suggested that the the information gathered below should be completed in a quick 15 minute discussion with relevant treating OT and PT therapists on the ward going through the ward list. We would suggest marking up on the patient list the following information against each patient to assist with collating the below information: active OT and PT involvement, presence of a self-mangagement program (for control wards) or My Therapy Program (for wards who have crossed over to the My Therapy conditions) from OT and/or PT.

Please emphasise that usual care should not change for the control wards.

Date ______________________

Has the ward transitioned across to My Therapy?
◯ Yes
◯ No

On the day this audit is completed, what is the number of occupied beds on ward? ______________________

How many beds are on the ward? ______________________

How many of these patients have active OT or PT involvement and presence of a self-management program (for wards transitioned across to My Therapy, this would be the My Therapy), that meet the following criteria:
- A written program (delivered either electronically or on paper)
- Documented in the medical record
- Actively monitored and progressed as clinically required
- Have a feedback mechanism to the therapist (e.g. through a paper based or smart device tick sheet or verbal feedback to therapist which has been documented in the medical history)
***If it does not meet all of these criteria, it is advice/education not a self-management program.**

	Active OT involvement	Active PT involvement	Presence of a self-management program / My Therapy Program from OT	Presence of a self-management program / My Therapy Program from PT

Bed/Patient 1	☐	☐	☐	☐
Bed/Patient 2	☐	☐	☐	☐
Bed/Patient 3	☐	☐	☐	☐
Bed/Patient 4	☐	☐	☐	☐
Bed/Patient 5	☐	☐	☐	☐
Bed/Patient 6	☐	☐	☐	☐
Bed/Patient 7	☐	☐	☐	☐
Bed/Patient 8	☐	☐	☐	☐
Bed/Patient 9	☐	☐	☐	☐
Bed/Patient 10	☐	☐	☐	☐
Bed/Patient 11	☐	☐	☐	☐
Bed/Patient 12	☐	☐	☐	☐
Bed/Patient 13	☐	☐	☐	☐
Bed/Patient 14	☐	☐	☐	☐
Bed/Patient 15	☐	☐	☐	☐
Bed/Patient 16	☐	☐	☐	☐
Bed/Patient 17	☐	☐	☐	☐
Bed/Patient 18	☐	☐	☐	☐
Bed/Patient 19	☐	☐	☐	☐
Bed/Patient 20	☐	☐	☐	☐
Bed/Patient 21	☐	☐	☐	☐
Bed/Patient 22	☐	☐	☐	☐
Bed/Patient 23	☐	☐	☐	☐
Bed/Patient 24	☐	☐	☐	☐
Bed/Patient 25	☐	☐	☐	☐
Bed/Patient 26	☐	☐	☐	☐
Bed/Patient 27	☐	☐	☐	☐
Bed/Patient 28	☐	☐	☐	☐
Bed/Patient 29	☐	☐	☐	☐
Bed/Patient 30	☐	☐	☐	☐
Bed/Patient 31	☐	☐	☐	☐
Bed/Patient 32	☐	☐	☐	☐
Bed/Patient 33	☐	☐	☐	☐
Bed/Patient 34	☐	☐	☐	☐
Bed/Patient 35	☐	☐	☐	☐
Bed/Patient 36	☐	☐	☐	☐
Bed/Patient 37	☐	☐	☐	☐
Bed/Patient 38	☐	☐	☐	☐
Bed/Patient 39	☐	☐	☐	☐
Bed/Patient 40	☐	☐	☐	☐

Subacute Program (IPR or GEM) Admission FIM - Motor score Admission FIM - Cognitve score

Bed / Patient 1 ______ ______ ______
Bed / Patient 2 ______ ______ ______
Bed / Patient 3 ______ ______ ______
Bed / Patient 4 ______ ______ ______
Bed / Patient 5 ______ ______ ______
Bed / Patient 6 ______ ______ ______
Bed / Patient 7 ______ ______ ______
Bed / Patient 8 ______ ______ ______
Bed / Patient 9 ______ ______ ______
Bed / Patient 10 ______ ______ ______
Bed / Patient 11 ______ ______ ______
Bed / Patient 12 ______ ______ ______
Bed / Patient 13 ______ ______ ______
Bed / Patient 14 ______ ______ ______
Bed / Patient 15 ______ ______ ______
Bed / Patient 16 ______ ______ ______
Bed / Patient 17 ______ ______ ______
Bed / Patient 18 ______ ______ ______
Bed / Patient 19 ______ ______ ______
Bed / Patient 20 ______ ______ ______
Bed / Patient 21 ______ ______ ______
Bed / Patient 22 ______ ______ ______
Bed / Patient 23 ______ ______ ______
Bed / Patient 24 ______ ______ ______
Bed / Patient 25 ______ ______ ______
Bed / Patient 26 ______ ______ ______
Bed / Patient 27 ______ ______ ______
Bed / Patient 28 ______ ______ ______
Bed / Patient 29 ______ ______ ______
Bed / Patient 30 ______ ______ ______
Bed / Patient 31 ______ ______ ______
Bed / Patient 32 ______ ______ ______
Bed / Patient 33 ______ ______ ______
Bed / Patient 34 ______ ______ ______
Bed / Patient 35 ______ ______ ______
Bed / Patient 36 ______ ______ ______
Bed / Patient 37 ______ ______ ______
Bed / Patient 38 ______ ______ ______
Bed / Patient 39 ______ ______ ______
Bed / Patient 40 ______ ______ ______

Supplementary file 3: Staff Survey

My Therapy Sustainability Survey

Project Title My Therapy
Project ID Number 69610
Project Sponsor
Monash University
Principal Investigators
1. Dr Natasha Brusco, Monash University
2. Professor Natasha Lannin, Monash University and Alfred Health
3. Professor Meg Morris, La Trobe University and Healthscope
4. Professor Nick Taylor La Trobe University and Eastern Health
5. Katie Dixon, Cabrini Health
Other Investigators 6. Dr Christina Ekegren, Monash University and Alfred Health
7. Ms Sara Whittaker, Monash University and Eastern Health

Introduction
You are invited to take part in this research project about My Therapy because you are currently an Occupational Therapist or a Physiotherapist working in a setting which has provided My Therapy to patients. Your contact details were obtained through your employment at Alfred Health, Cabrini Health, Eastern Health or Healthscope.

Are you currently working as a registered Occupational Therapist or a Physiotherapist at Alfred Health, Cabrini Health, Eastern Health or Healthscope?

If no, thank you for your time. Unfortunately, to be included in this study you must be currently working as a registered Occupational Therapist or a Physiotherapist at Alfred Health, Cabrini Health, Eastern Health or Healthscope.

◯ Yes
◯ No

Benefits of the Project
The findings of this project will improve our understanding of rehabilitation using My Therapy and how to best provide Allied Health services to people within rehabilitation settings. The information will be directly beneficial in advocacy and policy development for rehabilitation patients.

Participant Involvement, Confidentiality and Anonymity
Occupational Therapists and Physiotherapists who agree to participate in the research are asked to complete this online questionnaire, which should take less than 20 minutes of your time.

Participation in the research is completely voluntary and you may, without any penalty,
decline to take part or withdraw your response at any time without providing an explanation, or refuse to answer a question. Your ongoing employment will not be compromised by your decision to participate or not in the research. Your answers will be completely anonymous and confidential. The only potential risks to participation relate to privacy and confidentiality. Please be assured that all the data collected from you will be stored securely and only accessed by the research team.
The research outcomes may be presented at conferences and written up for publication. However, in all these reports, the privacy and confidentiality of individuals will be protected.

Data storage

All information will be stored securely on a password-protected computer database on the Monash University server. All information will be kept for seven years. After this time, all information (electronic and hard copy) will be destroyed. All participants have the right to access, and request correction of, their information in accordance with the Freedom of Information Act 1982 (Vic.).

Ethics Committee Clearance
The ethical aspects of this research project have been approved by the Alfred Hospital Ethics Committee, Monash University Human Research Ethics Committee (HREC), Eastern Health HREC and the La Trobe University HREC. Cabrini Research Governance Office has approved the conduct of this study at Cabrini Health.

This project will be carried out according to the National Statement on Ethical Conduct in Human Research 2007 produced by the National Health and Medical Research Council of Australia. This statement has been developed to protect the interests of people who agree to participate in human research studies.

Queries and concerns
The person you may need to contact will depend on the nature of your query, as follows:

a) For further information:

If you want any further information concerning this project, if you wish to withdraw or if you have any problems which may be related to your involvement in the project, you can contact the following person:

Name Christina Ekegren
Position Project Manager, My Therapy
Telephone 0447 011 876
Email christina.ekegren@monash.edu

b) For complaints:

If you have any complaints about any aspect of the project, the way it is being conducted or any questions about being a research participant in general, then you may contact:

Name Complaints Officer
Position Office of Ethics & Research Governance, The Alfred

Telephone (03) 9076 3619

Email research@alfred.org.au

Please quote the following Project ID Number: 69610

If you decide to continue, you are consenting to take part in the survey.

Thank you for agreeing to complete this survey. This survey considers the use of My Therapy within your clinical practice at present.

Date ______________________________

QUESTION 1: Are you working as an Occupational Therapist or Physiotherapist?

◯ Occupational Therapist
◯ Physiotherapist

QUESTION 2: Gender

◯ Male
◯ Female
◯ Non-binary / Do not wish to disclose

QUESTION 3: How many years of clinical experience have you had?

◯ Less then one year
◯ Between 1-2 years
◯ Between 3-4 years
◯ Between 5-9 years
◯ Between 10-14 years
◯ Between 15-19 years
◯ Between 20-24 years
◯ More than 25 years

QUESTION 4: Where are you employed?

If you are employed across two health networks, please indicate this through your selection

☐ Alfred Health
☐ Cabrini Health
☐ Eastern Health
☐ Healthscope

QUESTION 5: How long have you been working on your current rehabilitation ward?

☐ Less then one year
☐ Between 1-2 years
☐ Between 3-4 years
☐ Between 5-9 years
☐ Between 10-14 years
☐ Between 15-19 years
☐ Between 20-24 years
☐ More than 25 years

Your current rehabilitation ward was involved in a clinical trial of My Therapy between April 2021 and April 2022.

My Therapy involves the self-management of activities and exercises, outside of supervised therapy, with rehabilitation patients as a way of increasing the amount of practice undertaken.

The guidelines for prescribing My Therapy are as follows:

The program must:

- Be in written form (delivered to the patient electronically or on paper);
- Be documented in the medical record;
- Be actively monitored and progressed as clinically required; and
- Have a feedback mechanism to the therapist (e.g. paper based or verbal feedback, documented in the medical

record)

QUESTION 6: Were you employed on the ward during this time?	◯ Yes<br>◯ No
QUESTION 7: During that time, did you ever prescribe a My Therapy program to a patient?	◯ Yes<br>◯ No
QUESTION 8: Do you provide patients with a My Therapy program as part of your usual care as an occupational therapist or physiotherapist?	◯ None of the time<br>◯ Rarely<br>◯ Some of the time<br>◯ All the time
QUESTION 9: On the occasions that you don't provide a My Therapy program, what are the MAIN reason(s) for this (thinking about your current patient list)?<br><br>Select all that are relevant to you	☐ Not considered part of usual care<br>☐ Admission assessment not yet completed, therefore too early in admission<br>☐ Patients medically unwell<br>☐ Not enough clinician time<br>☐ Family involvement required and not available<br>☐ Not a priority<br>☐ Other<br>☐ N/A
If other, please describe:	__________________________________
QUESTION 10: Do you know how to develop and deliver a My Therapy program?	◯ Yes<br>◯ No<br>◯ Unsure
If yes, what guides you to develop a patient's My Therapy program?	☐ My Therapy Program guidelines<br>☐ Internal health service policies and procedures<br>☐ External guidelines<br>☐ Peer learning from other therapists<br>☐ I don't follow any formal policies / guidelines, instead I use my own decision making<br>☐ Other
If other, please describe:	__________________________________
QUESTION 11: Have you been formally trained in delivering a My Therapy Program?	◯ Yes<br>◯ No<br>◯ Unsure
QUESTION 12: Have the objectives and your role in My Therapy been clearly defined?	◯ Yes<br>◯ No<br>◯ Unsure
QUESTION 13: As an occupational therapist or physiotherapist, do you feel it is part of your job to deliver My Therapy following the guidelines?	◯ Yes<br>◯ No<br>◯ Unsure
QUESTION 14: How confident are you that you have the skills to deliver My Therapy following the guidelines?	Not at all confident — Somewhat confident — Very confident<br>[scale]<br>*(Place a mark on the scale above)*

QUESTION 15: How confident are you that you can deliver My Therapy following the guidelines even when other professionals with whom you work with do not do this?

Not at all confident — Somewhat confident — Very confident

(Place a mark on the scale above)

QUESTION 16: How confident are you that you can deliver My Therapy following the guidelines even when there is little time?

Not at all confident — Somewhat confident — Very confident

(Place a mark on the scale above)

QUESTION 17: Please respond to this statement -

For me, delivering My Therapy following the guidelines is:

Not worthwhile at all — Somewhat worthwhile — Very worthwhile

(Place a mark on the scale above)

QUESTION 18: Will you deliver My Therapy following the guidelines in the next three months?

- ◯ Yes
- ◯ No
- ◯ Unsure

QUESTION 19: Do you think it is possible to tailor My Therapy programs to patients' needs?

- ◯ Yes
- ◯ No
- ◯ Unsure

QUESTION 20: To what extent do you agree with the following statement: My Therapy programs help patients to be more physically active?

Strongly disagree — Strongly agree

(Place a mark on the scale above)

QUESTION 21: To what extent do you agree with the following statement: Patients who receive a My Therapy program are motivated.

Strongly disagree — Strongly agree

(Place a mark on the scale above)

QUESTION 22: How confident are you that you can deliver My Therapy following the guidelines even when patients are not motivated?

Not at all confident — Somewhat confident — Very confident

(Place a mark on the scale above)

QUESTION 23: Compared to supervised therapy sessions, how important are My Therapy programs?

Much less important — Equally important — Much more important

(Place a mark on the scale above)

Question 24: In general, do you think My Therapy programs are compatible with 'usual care'?

- ◯ Yes
- ◯ No
- ◯ Unsure

QUESTION 25: In general, do you think that My Therapy programs are easy to deliver?

- ◯ Yes
- ◯ No
- ◯ Unsure

QUESTION 26: In the organisation you work, do you think that all necessary resources are available to deliver My Therapy programs?	◯ Yes<br>◯ No<br>◯ Unsure
QUESTION 27: Do you think delivering a My Therapy program following the guidelines is something you do automatically?	◯ Yes<br>◯ No<br>◯ Unsure
QUESTION 28: Do you believe My Therapy is now a part of usual care on your ward?	◯ Yes<br>◯ Somewhat<br>◯ No<br>◯ Unsure
QUESTION 29: Can you think of any strategies that have helped sustain My Therapy on the ward?	☐ Central auditing of My Therapy provision<br>☐ Central auditing of outcomes<br>☐ Patient experience surveys of My Therapy<br>☐ Therapist feedback of My Therapy to health service management<br>☐ Use of blue folders for My Therapy Programs<br>☐ Having my health service's logo on the My Therapy brochure<br>☐ Access to 'exercise' equipment ongoing<br>☐ Maintenance of exercise/task library on PTX/other location<br>☐ Having a staff member encourage us to support ongoing implementation of My Therapy (i.e. a 'local champion')<br>☐ Availability of My Therapy training materials<br>☐ Encouragement from senior staff/management to provide My Therapy programs<br>☐ Formal orientation to My Therapy (face to face)<br>☐ Formal orientation to My Therapy (online)<br>☐ My Therapy Mandatory Training (periodic)<br>☐ My Therapy Mandatory Training (once off)<br>☐ Online learning platform for My Therapy<br>☐ Increasing multi-disciplinary team knowledge of My Therapy<br>☐ Discussing My Therapy Program in case conference<br>☐ Discussing My Therapy Program in team meetings<br>☐ Engagement of other disciplines in My Therapy provision<br>☐ Involvement of multi-disciplinary team members in regular My Therapy training<br>☐ Development of local policies and procedures at individual health services regarding My Therapy<br>☐ Engagement of Allied Health Translation leads in sustaining My Therapy at the health services<br>☐ Collaboration with onward services (such as CRP) for continued My Therapy use<br>☐ Engagement of interested therapists/services in other areas/wards of the health services to support My Therapy wider rollout<br>☐ Other
Other (please describe)	____________________________________
QUESTION 30: Can you think of anything that has made it challenging to sustain My Therapy on the ward?	____________________________________

QUESTION 31: Do you have any comments to add about My Therapy in general?

Thank you for your responses!
